# Sex, Age, and Emotional Valence: Revealing Possible Biases in the ‘Reading the Mind in the Eyes’ Task

**DOI:** 10.3389/fpsyg.2018.00570

**Published:** 2018-04-24

**Authors:** Jana Kynast, Matthias L. Schroeter

**Affiliations:** ^1^Department of Neurology, Max Planck Institute for Human Cognitive and Brain Sciences, Leipzig, Germany; ^2^Day Clinic for Cognitive Neurology, University Hospital Leipzig – University of Leipzig, Leipzig, Germany

**Keywords:** theory of mind, social cognition, gender bias, mind reading, language of the eyes

## Abstract

The ‘Reading the Mind in the Eyes’ test (RMET) assesses a specific socio-cognitive ability, i.e., the ability to identify mental states from gaze. The development of this ability in a lifespan perspective is of special interest. Whereas former investigations were limited mainly to childhood and adolescence, the focus has been shifted towards aging, and psychiatric and neurodegenerative diseases recently. Although the RMET is frequently applied in developmental psychology and clinical settings, stimulus characteristics have never been investigated with respect to potential effects on test performance. Here, we analyzed the RMET stimulus set with a special focus on interrelations between sex, age and emotional valence. Forty-three persons rated age and emotional valence of the RMET picture set. Differences in emotional valence and age ratings between male and female items were analyzed. The linear relation between age and emotional valence was tested over all items, and separately for male and female items. Male items were rated older and more negative than female stimuli. Regarding male RMET items, age predicted emotional valence: older age was associated with negative emotions. Contrary, age and valence were not linearly related in female pictures. All ratings were independent of rater characteristics. Our results demonstrate a strong confound between sex, age, and emotional valence in the RMET. Male items presented a greater variability in age ratings compared to female items. Age and emotional valence were negatively associated among male items, but no significant association was found among female stimuli. As personal attributes impact social information processing, our results may add a new perspective on the interpretation of previous findings on interindividual differences in RMET accuracy, particularly in the field of developmental psychology, and age-associated neuropsychiatric diseases. A revision of the RMET might be afforded to overcome confounds identified here.

## Introduction

‘*Where words are restrained, the eyes often talk a great deal*’. The famous saying by Samuel Richardson (1689 – 1761) outlines the importance of human gaze as source of social and emotional information. Especially in ambiguous situations, non-verbal communication through gaze can hint at what to expect from another person. For instance, reading the eyes of the handsome person next to you facilitates to state if the attraction is mutual or if you’re about to get the brush-off. Subtle social cues appear to be gathered by a fine-tuned sensor, and are carefully processed further. The ability to adequately identify mental states of another person based on the perception and interpretation of such cues is termed ‘Theory of Mind’ (ToM). Information about another person’s mental state facilitates successful social interaction as it allows to predict his/her future behavior and, consequently, to react properly.

The ‘Reading the Mind in the Eyes’ test (RMET; Baron-Cohen et al., 1997, 2001) is one of the most frequently used tools for ToM assessment in adults (Kirkland et al., 2013; cf. Oakley et al., 2016), and can be applied to identify individuals with ToM impairment (Olderbak et al., 2015). The test has been originally designed in 1997 and revised in 2001. The revised version (Baron-Cohen et al., 2001) contains 36 pictures showing the eyes of men and women. Each item is presented with four adjectives describing mental states. Subjects are asked to select the word that matches the mental state of the person by evaluating her/his eyes only. According to research, sex (review by Vellante et al., 2013; meta-analysis by Kirkland et al., 2013), age (Pardini and Nichelli, 2009; Baglio et al., 2012; El Haj et al., 2015), and psychopathology (e.g., Asperger syndrome in Baron-Cohen et al., 1985, 1997, 2015, or behavioral variant frontotemporal dementia in Schroeter et al., 2014, 2018) are crucial factors that influence test performance.

The RMET is well-established and its psychometric properties have been investigated recently (e.g., Olderbak et al., 2015). The test contains an equal number of male and female photographs (18 each) that are standardized in size and that show identical parts of the eye-region (Baron-Cohen et al., 1997). Beyond these features, pictures are only insufficiently standardized (Hallerbäck et al., 2009; Olderbak et al., 2015). Details on selection criteria and the consideration of further item attributes are scarce (Baron-Cohen et al., 1997, 2001). In particular, information about the age range of the pictured persons is completely missing. Further, the test may also be gender biased, as female RMET stimuli often express ambiguous mental states and ‘sexual interest […] toward the viewer’ (Alvarez, 2013). The RMET photographs originate magazines (Baron-Cohen et al., 1997, 2001), which is why female items appear to be highly selective, representing model-like and made-up women. The source of pictures may cause another limitation of the test: solely Caucasian people are represented in the RMET. Although the concept of mind reading from the eyes seems universal (Adams et al., 2010), the application of the RMET is restricted to a defined cultural context due to a missing variety of ethnic groups.

Emotional valence influences perception and attention (e.g., Ebner et al., 2011). Consequently, this important attribute should be considered when constructing psychological tests assessing mental state recognition. For the revised RMET (Baron-Cohen et al., 2001), authors ensured that target words and distractors of each photograph have comparable emotional qualities, which was not the case in the original version (Baron-Cohen et al., 1997). However, it is not reported if male and female eyes convey similar emotional content (cf. Baron-Cohen et al., 2001). Two studies have tried to categorize the emotional spectrum of the RMET stimuli. In the study of Harkness et al. (2005), twelve female students rated each photo (presented with the corresponding target word) on a 7-point scale (1 = very negative; 7 = very positive). The rating realized by this small sample of young, highly educated females revealed that the RMET stimuli convey mostly neutral and negative emotions (cf. **Figure 2A**). At the same time, positive emotions are overrepresented in female items. Further, the combined presentation of the photo with the target word may have biased the results as it is impossible to identify the primary source of information used for emotional valence rating (eyes vs. verbal information). Yet, this classification scheme was often used in other studies (e.g., Maurage et al., 2011). More recently, Konrath et al. (2013) classified emotional valence of 17 RMET items based on information from ‘Whissell’s Dictionary of Affect on Language’ (Whissell, 2002). The target words of six items were categorized as positive, nine were rated negative and two were not classified as they were not listed in the dictionary (Konrath et al., 2013). Critically, this study did not classify the complete RMET, and completely neglected facial information which seems crucial for mind reading from the eyes (Konrath et al., 2013).

As personal attributes modulate cognitive processing when (mis-) matching characteristics of the beholder, the investigation of individual features of the persons depicted in the RMET is highly relevant. The RMET is supposed to assess the ability to attribute mental states from gaze and is often used in clinical settings but also recently to investigate socio-cognitive aging in normal elderly (Kynast et al., 2017). As already mentioned age and sex have been found to modulate RMET accuracy. It is thus worth investigating the structure of the stimulus attributes to verify (or possibly reject) the presumption that the stimulus set is appropriate to assess mind reading in adults across the lifespan. It is though possible that previously found inter-individual differences in RMET performance may have been triggered by an unequal distribution of emotional valence and age among male and female stimuli. In particular, similarity in age, sex and race can attract attention and lead to preferred perception and processing of human stimuli (Ebner et al., 2013). Especially when investigating the ability to perceive emotions at older age, it has been shown that the quality of emotions can also modulate attentional processes (e.g., Ebner et al., 2011). For instance, the so-called ‘positivity effect’ (Kennedy et al., 2004) describes the phenomenon that elderly preferably focus on positive emotions in their environment, while rejecting negative emotions more often. Specifically, if the RMET stimulus set included a larger number of items depicting neutral or negative emotions, this might be an explanation for a higher accuracy at younger age compared to advanced age shown in several studies. The possibility that the stimulus characteristics may modulate emotion perception and mental state recognition from the eyes has never been investigated before. Thus, the present study aims to provide information on essential characteristics of the persons pictured in the RMET as they potentially impact mental state recognition. In particular, it focusses on age, sex and emotional valence of the stimuli. For the first time, the age range of the RMET stimuli is subject of investigation. Further, the present study provides an estimate of the emotional valence spectrum of the RMET based on the sole evaluation of facial expression.

## Materials and Methods

### Materials and Procedure

Data was assessed in an online survey in the framework of citizen science, i.e., public participation in scientific research. All 36 pictures of the revised version of the RMET (Baron-Cohen et al., 2001) were presented without any verbal information on mental status (no target or distractor words), and no information on sex of the pictured person was given. For each photograph, participants were asked to estimate (1) its emotional valence ranging from 1 (very negative) to 10 (very positive) on a visual scale, and (2) the age of the pictured person. Age judgements should be typed via keyboard. Instructions were given in German and English. Participants’ age, sex and nationality were registered, while their identity remained unknown. The survey was self-guided and took approximately 15 min. for completion. The link to the online survey was shared via e-mail and social networks. This study was carried out in accordance with the recommendations of the ethics guidelines provided by the German Research Foundation (DFG) for psychological research with written informed consent from all subjects. All subjects gave written informed consent in accordance with the Declaration of Helsinki. The protocol was approved.

### Analysis Sample

43 individuals between 22 and 56 years completed the online survey (19 male, age 29.33 ± 6.86 years, mean ± standard deviation). Participation was voluntarily, anonymous and unpaid. All participants were informed about the objective, procedure and task of the online survey. Demographics included age, sex and nationality.

Male and female participants did not differ in age [*t*(41) = 0.45, *p* > 0.25]. Most participants were Europeans (35 German, one French, two Italian, two Spaniards, one Ukrainian), but also two US-citizens completed the survey. All answers were carefully checked for plausibility and a lack of variability in the answering pattern, as this would indicate non-conformity to task instructions and/or social cognitive deficits. All participants were included in data analysis.

### Statistical Analyses

**FIGURE 1:**
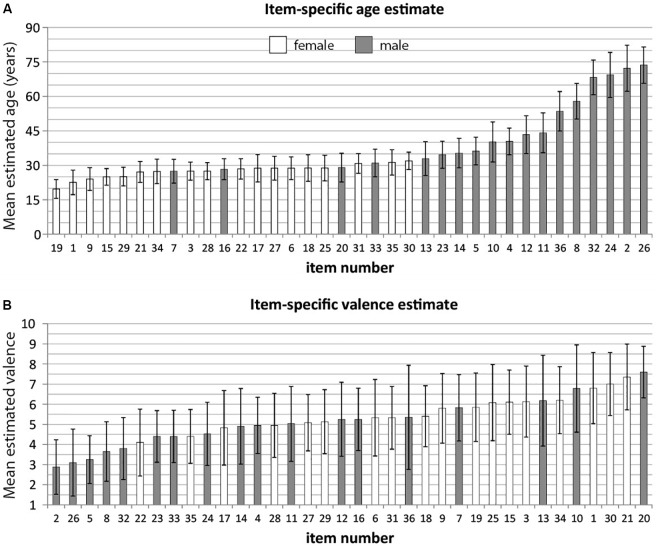
**(A)** Mean estimated age (*N* = 43) and standard deviation for each ‘Reading the Mind in the Eyes’ test (RMET) item. Items are sorted in ascending order according to mean estimated age; items are numbered according to their appearance in the RMET. Male items are marked dark, female items are marked white. **(B)** Mean estimated valence (*N* = 43) and standard deviation for each RMET item. Items are sorted in ascending order according to mean estimated valence; items are numbered according to their appearance in the RMET. Male items are marked dark, female items are marked white.

Mean ratings of age and valence were computed for each item over the whole sample (**Figures 1**, **3**). Variables were visually checked for normal distribution. Age ratings were not normally distributed among the items. Therefore, this variable was log-transformed, and normal distribution was given thereafter.

**FIGURE 2:**
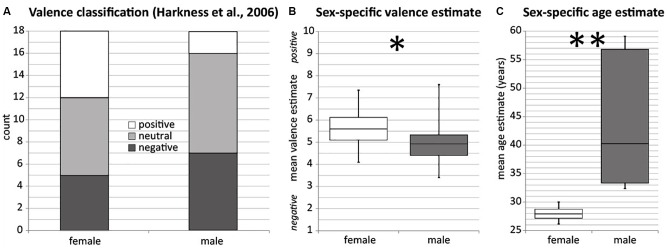
**(A)** Number of male and female items classified as positive, neutral, and negative according to Harkness et al. (2005). **(B)** Mean estimated valence over all male (dark) and female (white) RMET items. Higher scores correspond to positive emotional valence. Independent sample *t*-test yielded significant group differences. **(C)** Mean estimated age over all male (dark) and female (white) RMET items. Mann–Whitney-*U*-test yielded significant group differences. ^∗∗^*p* < 0.001 ^∗^*p* < 0.05.

**FIGURE 3:**
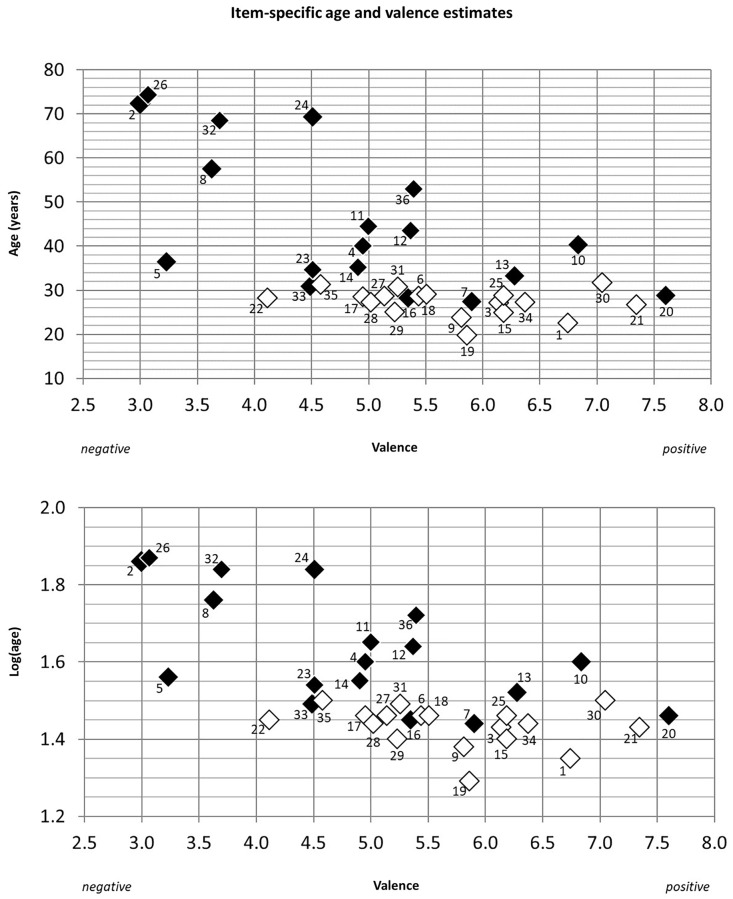
Age and valence distribution for male (dark squares) and female subjects (white squares). Age is shown in years, additionally log transformed to correspond to the performed statistical analyses. Items are numbered according to their appearance in the RMET. Higher valence scores correspond to positive emotional valence.

Firstly, the interrelation between item characteristics was analyzed. An independent sample *t*-test tested differences in emotional valence ratings between male and female items. Parametric analysis of age-differences between male and female items was not applicable, as the variances between groups in both age and log(age) were not homogeneous. Thus, the non-parametric Mann–Whitney-*U*-test was applied. For both age and valence, Hedges g was computed as an effect size measure. Hedges g was chosen as it is supposed to be more precise than Cohen’s d when sample sizes are rather small (*n* < 20; Durlak, 2009). Secondly, the relation of rater characteristics on valence and age ratings was tested. Two separate linear regression analyses were applied to predict the item’s (1) log(age) and (2) valence from rater age and sex. Thirdly, the linear relation between emotional valence and log(age) was tested separately (1) over all items, (2) for male items only, and (3) for female items only with linear regression. This was done to disentangle a possible interaction between both factors.

## Results

**Figure 1** gives an overview about item-specific estimates of age (**Figure 1A**) and valence (**Figure 1B**). The estimated age ranged from 19.68 to 73.63 years. The estimated age distribution had a greater variance for male (45.4 ± 16.23 years, range 27–74) compared to female stimuli (27.21 ± 3.07 years, range 19–31), which was statistically significant (Levene’s Test: *F*(1,34) = 31.8, *p* < 0.001). **Figure 2C** depicts the mean estimated age of male and female items. Group-differences were therefore tested non-parametrically. Male items were rated significantly older compared to female items [*U*(*n*_male_ = 18, *n*_female_= 18) = 25, *p* < 0.001]. Using females as reference group, the resulting effect size g = 1.52 (95% confidence interval: 0.78–2.26) can be interpreted as a large effect (Cohen, 1988) reflecting younger age for female stimuli.

Regarding emotional valence, male items appeared to have a larger variance (4.87 ± 1.28, range 3–7.6), while female eyes represented a narrow emotional range (5.72 ± 0.85, range 4.11–7.34). However, the difference in variance between male and female items as tested with Levene’s test was not significant [*F*(1,34) = 1.84, *p* = 0.184], justifying the application of parametric tests for the comparison of the group means. An independent sample *t*-test revealed significant valence differences [*t*(34) = 2.33, *p* = 0.026]. Female items were rated more positive than male items. Using males as reference group, an effect size of *g* = 0.76 (95% confidence interval: 0.09–1.44) can be interpreted as medium-sized effect reflecting more positive emotions among female stimuli (Cohen, 1988). **Figure 2** contrasts the sex-specific valence classification by Harkness et al. (2005; **Figure 2A**) with the current results (**Figure 2B**). In both studies, male items lack positive emotions compared to neutral and negative emotions.

Linear regression analyses predicting log(age) and emotional valence from rater sex and age yielded no significant results. Neither emotional valence [*F*(2,40) = 2.09, *p* = 0.137] nor log(age) [*F*(2,40) = 1.2, *p* > 0.25] were linearly associated with rater characteristics. The interrelation between item sex and estimates of age and emotional valence are visualized in **Figure 3**. Linear regression of log(age) on emotional valence over all items revealed a significant negative relation between the variables [*F*(1,34) = 21.32, *p* < 0.001, *R*^2^ = 0.385]. The regression equation can be defined as *y* = 12.65–4.79^∗^x. Due to the log-transformation of the independent variable age, the relation between age (x) and valence (y) can be approximated by the formula Δ_y_ ≈ (β_x_/100) ^∗^ %Δ_x_. Thus, comparing two stimuli with different age estimates, a relative change in age of ten percent is related to a decrease in estimated valence of 0.48 points (scale range 1–10). For instance, a person at the age of 30 years is rated approximately 2.39 points less positive than a person at the age of 20^1^.

In male items, emotional valence was strongly predicted by estimated age [*F*(1,16) = 10.65, *p* = 0.005, *R*^2^ = 0.4]. The regression equation is defined as *y* = 13.78–5.46^∗^x. As the regression coefficient of log(age) is -5.46 for male eyes, a relative change in age of ten percent is related to a decrease in estimated valence of 0.5 (scale range 1–10). Thus, a 30-year-old male photo is rated approximately 2.5 points less positive compared to a 20-year-old man^1^, while an 80-year-old man is rated 1.5 points less positive compared to a 60-year-old man^2^. When analyzing female items only, no significant linear relation was obtained [*F*(1,16) = 1.01, *p* = 0.062, *R*^2^ = 0.06]. Thus, the overall relation between age and emotional valence is driven by male items only, whereas ratings of age and emotional valence are not linearly associated in female items. Note that analyses of the German participants alone yielded comparable results.

## Discussion

This study evaluates the interrelatedness of stimulus attributes of the RMET, one of the most frequently used instruments for ToM assessment in adults. In this study, 43 persons estimated age and emotional valence of the RMET photos picturing the eye-region of a person. To our knowledge, this is the first study assessing estimates of age for the RMET pictures, and relating it to the person’s sex and emotional quality. Harkness et al. (2005) provided an often cited emotional classification scheme for the RMET stimuli. The methodology of our work varies in certain aspects: our sample contains more participants, and is more diverse with respect to age and sex, while the ethnic background is similar. Further, rating was solely based on the evaluation of eye gaze in our study, as no verbal content was presented in the current study. With this, we avoid a potential impact of verbal information on emotion recognition and emotional valence rating. Additionally, the disengagement of verbal content allows the generalizability of our results for similar cultural backgrounds and independent of language.

Our results show that age and emotional valence are largely independent of rater attributes, indicating validity of the rating by precluding a specific rater bias. Interestingly, the estimated age range massively differed between male and female items. While female items were rated significantly younger than male items, the age range was strongly limited in the female item set. Conversely, male items presented a much broader variability in age, covering whole adult age range. Related to this, results clearly demonstrate that age and emotional valence are interrelated in the RMET. The older a person was rated, the less positive was the judgment of the emotional expression. However, this holds true for male items only: old men were rated more negative compared to young men. Contrary, the age range of female items is narrow, which is why age and emotional valence are not linearly related. Further, the variability in women’s emotional valence is strongly limited and slightly skewed towards positive emotions. Taken together, the RMET stimulus set does neither include women with a perceived age in the middle and late adulthood, nor do female eyes convey negative emotional content. Besides a lack of variability in the female’s emotion and age spectra, these attributes are strongly confounded in male items. These findings might mirror sex and age prejudices in the media, as the stimulus material originates from magazines.

Research has consistently demonstrated that individual characteristics, i.e., sex, age, race, and emotional valence, have a crucial impact on the salience of social information and its consecutive processing. Considering that the material used in the RMET is not balanced for these features, previously made conclusions about inter-individual differences in mind reading associated with stimulus attributes may be erroneous and need to be re-evaluated cautiously. In particular, differences in RMET accuracy in response to male versus female items (e.g., Schiffer et al., 2013) can no longer be explained by sex of the depicted person alone, as we demonstrate a strong interrelation of this feature with age and emotional valence. Since the RMET does not represent the full spectrum of emotional valence equally expressed by both sexes with a similar variety in age, this may explain the test’s low internal consistency (see Olderbak et al., 2015). Consequently, it is impossible to disentangle effects of stimulus attributes on mentalizing with the RMET. Specifically, the question if a lower accuracy in elderly compared to younger adults found in previous studies (e.g., Pardini and Nichelli, 2009; Baglio et al., 2012; El Haj et al., 2015) relates to age-related differences in the perception of emotions, i.e., the absence of the ‘positivity effect’ due to a lack of positive emotions (or an overweight of negative emotions) in the RMET stimulus set, may not be answered completely due to the confounding of emotional valence with sex and age of the depicted persons. For the investigation of specific research questions regarding inter-individual differences in mind reading that are related to sex, age, race, or emotional valence, alternative instruments may be applied.

Nevertheless, the RMET was constructed as an advanced test of ToM, as the stimuli provide only limited social information for mental state identification. Its major strength has always been the reliable distinction between individuals with and without ToM deficits specifically in the context of autism but also other psychiatric conditions. Thus, the RMET remains a powerful tool in clinical diagnostics. However, a revision of the test material may be necessary to investigate underlying mechanisms of social information perception and processing. This revision shall consider the potential weaknesses in stimulus selection, and aim towards a balance among relevant stimulus attributes such as sex, age, ethnicity, and quality of emotions. Our findings, which shall be confirmed in larger studies, may serve as a basis for the initiation of this revision process, as well as for the cautious re-interpretation of findings on inter-individual differences in RMET performance.

## Author Contributions

JK designed the work, acquired and analyzed the data, interpreted the results, and drafted the manuscript. MS designed the work, interpreted the results, and revised the manuscript draft critically for important intellectual content. All the authors approved the final version of the manuscript to be published and agreed to be accountable for all aspects of the work in ensuring that questions related to the accuracy or integrity of any part of the work are appropriately investigated and resolved.

## Conflict of Interest Statement

The authors declare that the research was conducted in the absence of any commercial or financial relationships that could be construed as a potential conflict of interest.

## Footnotes

1 Age difference 10 years (absolute)/50% (relative)

2 Age difference 20 years (absolute)/30% (relative)
